# Angiogenesis and Anti-Angiogenic Treatment in Prostate Cancer: Mechanisms of Action and Molecular Targets

**DOI:** 10.3390/ijms22189926

**Published:** 2021-09-14

**Authors:** Evangelia Ioannidou, Michele Moschetta, Sidrah Shah, Jack Steven Parker, Mehmet Akif Ozturk, George Pappas-Gogos, Matin Sheriff, Elie Rassy, Stergios Boussios

**Affiliations:** 1 Department of Paediatrics and Child Health, Chelsea and Westminster Hospital, 369 Fulham Rd., London SW10 9NH, UK; ioannidoueva@gmail.com; 2 CHUV, Lausanne University Hospital, Rue du Bugnon 21, CH-1011 Lausanne, Switzerland; michelemoschetta1@gmail.com; 3 Department of Medical Oncology, Medway NHS Foundation Trust, Windmill Road, Gillingham, Kent ME7 5NY, UK; sidrah.shah@nhs.net (S.S.); j.parker14@nhs.net (J.S.P.); 4 Department of Medical Oncology, Sisli Memorial Hospital, Kaptan Paşa Mah. Piyale Paşa Bulv., Okmeydanı Cd. 4, Istanbul 34384, Turkey; ozturkakif@yahoo.com; 5 Department of Surgery, University Hospital of Ioannina, 45111 Ioannina, Greece; pappasg8@gmail.com; 6 Department of Urology, Medway NHS Foundation Trust, Windmill Road, Gillingham, Kent ME7 5NY, UK; matin.sheriff@nhs.net; 7 Department of Cancer Medicine, Gustave Roussy Institut, 94805 Villejuif, France; elie.rassy@hotmail.com; 8 Faculty of Life Sciences & Medicine, School of Cancer & Pharmaceutical Sciences, King’s College London, London SE1 9RT, UK; 9 AELIA Organization, 9th Km Thessaloniki, Thermi, 57001 Thessaloniki, Greece

**Keywords:** prostate cancer, castration-resistant prostate cancer, hormone-sensitive prostate cancer, antiangiogenics, advances

## Abstract

Prostate cancer (PC) is the most common cancer in men and the second leading cause of cancer-related death worldwide. Many therapeutic advances over the last two decades have led to an improvement in the survival of patients with metastatic PC, yet the majority of these patients still succumb to their disease. Antiagiogenic therapies have shown substantial benefits for many types of cancer but only a marginal benefit for PC. Ongoing clinical trials investigate antiangiogenic monotherapies or combination therapies. Despite the important role of angiogenesis in PC, clinical trials in refractory castration-resistant PC (CRPC) have demonstrated increased toxicity with no clinical benefit. A better understanding of the mechanism of angiogenesis may help to understand the failure of trials, possibly leading to the development of new targeted anti-angiogenic therapies in PC. These could include the identification of specific subsets of patients who might benefit from these therapeutic strategies. This paper provides a comprehensive review of the pathways involved in the angiogenesis, the chemotherapeutic agents with antiangiogenic activity, the available studies on anti-angiogenic agents and the potential mechanisms of resistance.

## 1. Introduction

Prostate cancer (PC) is the most common cancer in men and the second most prevalent cause of cancer-related death worldwide [1]. Despite recent major breakthroughs in the treatment, PC remains a major public health concern, with more than 1.1 million cases worldwide detected every year. In the United Kingdom, over 47,000 men are annually diagnosed with PC and over 330,000 men are currently living with the disease [2].

A variety of genetic, hereditary and environmental factors have been proven to increase the risk of developing PC, such as older age, family history of PC and African ethnicity. The vast majority of patients present with non-specific and vague symptoms, such as decreased urinary stream, urgency, hesitancy, nocturia or incomplete bladder emptying; this is predominantly why it often presents at an advanced stage at diagnosis. Consequently, mortality is still relatively high with 26,730 estimated deaths in 2017 [3]. The diagnosis of PC is based on the microscopic evaluation of prostate tissue obtained via needle biopsy, performed with transrectal ultrasound guidance to obtain 10 to 12 tissue samples in a grid-like pattern. A primary Gleason grade is reported for the predominant histological pattern and a secondary grade for the highest pattern, based on the microscopic architecture and appearance of the cells. Tests for serum PSA variants estimate the probability of PC in patients with a previous negative biopsy [4,5]. The PC antigen 3 test is performed using urine collected after prostatic massage and has been validated in this population, demonstrating an 88% negative predictive value for subsequent biopsy [6]. New imaging technology has also been adapted to the diagnostic pathways. The most notable is magnetic resonance imaging (MRI), which uses a specialized phase in addition to T2-weighted imaging [7]. The reported sensitivity and specificity of MRI for identifying PC is 89% and 73%, respectively [8]. Targeted biopsies of suspicious lesions can then be obtained through either MRI image fusion with transrectal ultrasound using computerized software, or in-bore percutaneous biopsy during the actual MRI. Patients are stratified into low, intermediate and high risk, based on the sum of Gleason patterns, prostate-specific antigen (PSA) level and clinical stage [9,10,11]. The clinical utility of molecular and image-based biomarkers remains an area of active investigation, especially with concurrent updates to pathological risk stratification and PC treatment. Using biopsy tissue, a cell cycle progression score based on 31 genes can predict clinical progression and prostate cancer mortality [12]. Interest has grown in molecular or functional imaging with positron emission tomography (PET). Multiple radiotracers demonstrate activity in prostate cancer. Among them, three (C-choline, 18F-fluciclovine and 18F-sodium fluoride PET) have already received approval from the US Food and Drug Administration (FDA) [9,13]. Beyond these approved agents, use of PET-CT and PET-MRI compares favorably with existing modalities, particularly in patients with low PSA levels and for detection of regional lymph node metastases.

Patients with localized disease may be treated with active surveillance, surgery and radiation [14]. Active surveillance consists of series of PSA testing, physical examinations, prostate biopsies or a combination of these to monitor for disease progression [14]. Surgery and radiation continue to be effective treatments for patients with more advanced disease, such as those with a PSA level greater than 10 ng/mL and/or palpable nodules on digital rectal examination. With regards to surgery, open radical prostatectomy has been largely replaced with robotic radical prostatectomy, which is associated with better one-year urinary and sexual function outcomes compared with open surgery [15,16]. Radiation therapy has also undergone technological advances and intensity-modulated radiation therapy has mostly replaced 3D-conformal radiation. It delivers nonuniform radiation beams that can conform to irregularly shaped organs, which results in reduction of radiation to surrounding tissues [17,18]. Androgen deprivation therapy (ADT) is the cornerstone of the regimens used in the first-line treatment of patients with metastatic PC [19,20,21,22,23,24,25,26]. The demonstration of survival benefit using docetaxel-based therapy led to the approval by the FDA in 2004 of docetaxel and prednisone for the treatment of metastatic castration-resistant PC (mCRPC) [27]. Among newer agents, two act on the androgen axis; abiraterone acetate inhibits androgen biosynthesis, whereas enzalutamide, darolutamide and apalutamide interfere with androgen-receptor signaling. These therapies improve overall survival (OS) and secondary end points, such as skeletal-related events, pain and quality of life [19,20,21,22,23,24,25,26]. Sipuleucel-T, an autologous cellular immunotherapy, became the first FDA-approved cancer vaccine in the United States, increasing median OS by 4.1 months compared with placebo. This therapy is recommended for patients who are asymptomatic or minimally symptomatic and also when their PSA levels are low [22]. Cabazitaxel represents a tubulin-binding taxane, which increased median OS by 2.4 months compared with mitoxantrone [20]. Radioligand therapies, such as lutetium-177 (^177^Lu)–PSMA-617 can target PC cells, while sparing most normal tissues in patients selected with imaging to confirm radionuclide binding [26]. The addition of ^177^Lu-PSMA-617 to standard care significantly extended survival of patients with mCRPC, previously treated with androgen-receptor inhibitors and taxanes, who have recurrent disease. More recently, large-scale sequencing efforts have allowed for a better understanding of the genomic landscape of PC. Germline or somatic aberrations in the DNA damage repair genes are found in 19% of primary PC and almost 23% of mCRPC and compromise genomic integrity. DNA damage repair-targeting agents are being evaluated either as single agents or in combination with treatments eliciting DNA damage in clinical studies enrolling patients with PC [28].

Angiogenesis plays a major role in the development and spread of PC [29]. Among others, PC has the ability to produce matrix metalloproteinases (MMPs), vascular endothelial growth factor (*VEGF*), transforming growth factor β (*TGFβ*) and cyclooxygenase-2 (*COX-2*) [30,31,32,33]. The microenvironment of PC is a critical determinant of cancer onset and development [34]. Micro-vessel density (MVD), a measurement of PC angiogenesis, has been shown to be a predictor of metastasis and OS, and therefore targeting angiogenesis has been the subject of several clinical investigations and debates [35]. Moreover, the prognostic potential of angiogenic activity measurement holds great promise. This paper provides a comprehensive review of the pathways involved in the angiogenesis, the chemotherapeutic agents with antiangiogenic activity, the available studies on anti-angiogenic agents and the potential mechanisms of resistance.

### 1.1. Pathways Involved in the Angiogenesis of Prostate Cancer

**Figure 1:**
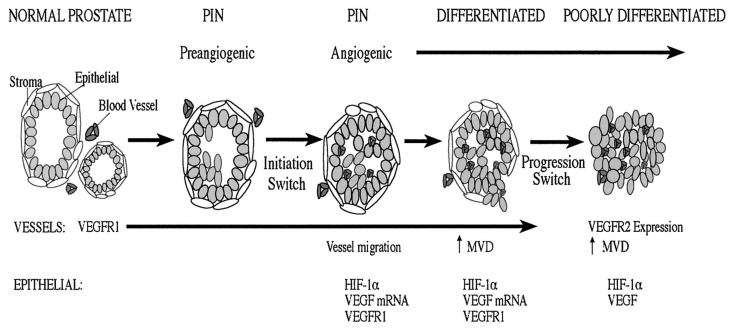
Normal prostate has prominent interductal vasculature, with VEGFR-1 expression. Preangiogenic epithelial neoplasia lesions lesions express VEGFR-1 and demonstrate a hypoxic environment that can stabilize HIF-1α. At this stage, the vasculature is interductal. Concomitant with epithelial neoplasia lesions, there is an angiogenic initiation switch that correlates with noticeable vessel migration into the prostatic duct, and the epithelial cells express HIF-1α. VEGF microRNA (miRNA) is expressed by the tumor cells and VEGFR-1 and protein are expressed by the tumor and endothelial cells. A second-event angiogenic progression switch is consistent with progression to a poorly differentiated tumor. In this environment, endothelial cells express VEGFR-2 and HIF-1α, and a detectable level of VEGF is expressed by tumor cells.

Angiogenesis is a complex multistep process involving endothelial cells, extracellular matrix and soluble factors. It is subdivided into several stages, including proteolytic degradation of the basement membrane and surrounding extracellular matrix, endothelial cell proliferation and migration and finally tube formation and structural reorganization. PC has the ability to produce angiogenic factors of which the most studied are reported in this section. The early angiogenic “initiation switch” correlates expression of hypoxia-inducible factor (HIF)-1α and VEGF tyrosine kinase receptor (VEGFR)-1 in addition to the recruitment and elaboration of intraductal vasculature in prostatic epithelial neoplasia lesions (Figure 1).

#### 1.1.1. Vascular Endothelial Growth Factors (VEGFs)

VEGF is the most prominent cytokine responsible for endothelial cell differentiation, migration, proliferation, tube formation, and vessel assembly. Among other functions, VEGF stimulates angiogenesis. It has five different isoforms that are generated by alternate splicing of a single gene: *VEGF-A*, *VEGF-B*, *VEGF-C*, *VEGF-D*, and *VEGF-E*. Research has mainly focused on VEGF-A, which is considered the most important form in cancer related neoangiogenesis. There are three VEGFR; VEGFR-1, -2 and -3, the first two of which bind to VEGF-A. Each VEGF family member binds with differential affinity for their receptors; for example, VEGFR-2 is primarily activated by VEGF-A and VEGFR-3 is only activated by VEGF-C and -D. Upon specific VEGF binding, the three VEGFR induce receptor dimerization and autophosphorylation leading to downstream signaling via a number of secondary messengers including several protein kinases and phosphatases that support a proangiogenic phenotype [36]. Important pathways include the phosphoinositide 3 kinase/protein kinase B/nuclear factor-kappa B (PI3K/Akt NF-kappaB) pathway that promotes cell survival, the mitogen-activated protein kinase (MAPK) pathway that promotes cell proliferation and the extracellular signal-regulated kinase (ERK) pathway that promotes cell proliferation, survival, differentiation, migration and angiogenesis. Through these signaling pathways, each of the VEGF family provides different actions. VEGF-A activation of VEGFR-2 represents the major mediator of angiogenesis induction in PC [37]. VEGF-induced endothelial cell proliferation and differentiation are mediated by the VEGFR-2 receptor. Considering that VEGF is involved in PC growth, some investigators supported its use as a prognostic marker for pre-therapeutic staging [38]. Several studies have demonstrated that there is a correlation between the *VEGF* expression in PC tissue and the VEGF plasma levels with the aggressive biological behavior of the disease [39,40,41]. The expression of *VEGF* and/or its receptor *VEGFR* in prostatic malignant tissue has also been shown to be directly associated with tumor Gleason grade, lymph node metastasis, and progression-free survival (PFS) [39,42]. One of the mechanisms that leads to lymph nodes metastases is the formation of lymphatic vessels within the tumor. *VEGF-C* is associated with lymph angiogenesis. There is evidence of significant association between *VEGF-C* expression and lymph node metastases in human PC cells, along with increased number of vessels expressing *VEGFR-3* [41]. Targeting VEGF-A has resulted in the first FDA drug approval of its class, Bevacizumab (Avastin; Genentech, Inc., South San Francisco, CA, USA) [43,44]. Aflibercept also targets the VEGF-A pathway by acting as a decoy receptor for VEGF-A. Sunitinib and cediranib are small multi-receptor molecule tyrosine kinase inhibitors with a demonstrated activity against VEGFR-1 and -2. Thalidomide is an immune-modulatory drug which also has anti-angiogenic effects. Lenalidomide is a more potent analogue of thalidomide with less prominent side effects. The mechanism of the anti-angiogenic effect of lenalidomide is not entirely elucidated but appears to be through multiple mechanisms, including inhibition of VEGF-induced phosphatidylinositol-3,4,5-trisphosphate (PI3K)-Akt pathway signaling [45].

#### 1.1.2. Fibroblast Growth Factors (FGFs)

Besides the VEGF family, the FGF group is a predominant growth factor family that possesses manifold roles on the process of PC progression. FGFs are potent mitogens to a plethora of cell types, such as endothelial cells, and are demonstrated in a variety of tissues where they majorly contribute to both physiological and pathological applications [46]. FGFs, particularly FGF2, FGF7 and FGF10, have a broad range of biological activities and participate in organogenesis, tissue homeostasis and the acquisition of androgen dependency [47]. PC cells and stromal cells in the PC microenvironment exude FGFs and convey FGF receptors (FGFRs) [48]. FGF1 and FGF2 were among the first identified angiogenic factors which promote angiogenesis during tumor growth. FGF/FGFR signaling regulates PC angiogenesis both in a VEGFA-dependent and independent manner [49]. Amplified FGF levels and FGFR articulation, such as type 1 FGFR (FGFR1), together with deviant FGFR signaling and the mislaying of the inherent FGF7/FGF10-type 2 FGFR (FGFR2), are connected to increased PC development and angiogenesis [49]. The serum basic FGF (bFGF) level has also been proved to be increased in PC patients. In addition, the relationship between FGF8 levels and VEGFA has been outlined to be correlated to progressed disease status, increased serum PSA results and poor prognosis [50]. These studies on the FGF/FGFR signaling cascade form the basis of FGF/VEGFR dual inhibition as a therapeutic strategy in PC. However, the utilization of FGF as a prognostic marker is debatable, as many research studies and reports have been inefficient in proving any correlation between the *FGF* expression and PC disease stage [51].

#### 1.1.3. Matrix Metalloproteinases (MMPs)

MMPs shape the extracellular matrix composition and influence the neoangiogenic process in a positive and negative directions. Metalloproteinases are zinc-containing calcium-dependent endopeptidases belonging to the metzincin superfamily [52]. MMPs were initially recognized for their contribution to cancer progression and metastasis, as they play a major role in destroying the connective tissue, therefore potentially increasing the chance of cancer cell metastasis. However, they are increasingly recognized as angiogenesis regulators as they have the ability to authorize endothelial cells attachment/detachment to the extracellular matrix, and therefore assist in endothelial cells migration and invasion [53]. The tissue inhibitors of metalloproteinases (TIMPs) regulate the MMP activity, and a disparity in the expression of MMPs and TIMPs has been depicted in PC angiogenesis [54]. Research has shown a higher MMPs to TIMPs proportion in progressive PC tumors (Gleason score of 8 and above) in comparison to tumors with a better prognosis (Gleason score of less than 6). The most commonly viewed members of the metalloproteinase group in PC are MMP-2, -7, -9 and membrane-type-1 matrix metalloproteinases (MT1-MMP) while, in more detail, MMP-2, MMP-7 and MMP-9 have been shown to stimulate PC angiogenesis [55].

#### 1.1.4. Transforming Growth Factor β (TGFβ)

TGFβ is a molecule with multiple phenotypic expressions and is composed of three isoforms, manifesting powerful tumor mutant characteristics in the initial stages of cancer development, whilst simultaneously bearing a tumor-promoting activity in the later stages of tumor progression [56]. This contradictory identity of TGFβ in PC is mostly because of its capacity to operate the extracellular signal-regulated kinase/mitogen-activated protein kinase (ERK/MAPK) activation in benign and malignant PC cells [57]. While there is no clear understanding of the exact point of when *TGFβ* stops being a tumor suppressor and becomes a tumor promoting factors, studies mainly have shown that it acts at the stromal-epithelial level via three different TGFβ receptors—TGFβ1, TGFβ2, and TGFβ3 present on tumor cells, as well as on nonmalignant stromal cells such as fibroblasts and endothelial cells [58]. Great amounts of TGFβ1 in PC tissues and elevation of TGFβ1 in the urinary and serum specimen of PC patients have been described to be connected to increased angiogenesis, metastasis, and poor clinical results [59]. TGFβ concomitantly influences PC angiogenesis through the overexpression of VEGFA by activating the SMAD-mediated transcriptional control and the Src/Focal Adhesion Kinase (FAK)/Protein kinase B (PKB or AKT) signaling [60]. Moreover, TGFβ also manages PC angiogenesis by enhancing the disparity of cancer-associated fibroblasts (CAFs), which subsequently promotes tumor angiogenesis via intensifying VEGFA development [59]. Moreover, VEGFA also regulates TGFβ expression by a positive feedback loop [61]. However, there are studies that have shown a negative correlation between TGFβ and VEGFA, especially in endothelial cells [58]. Finally, several strategies targeting TGF-β signaling by blocking integrin-mediated TGF-β activation were developed in preclinical models. Indeed, antibodies blocking integrins may impair the growth of primary and secondary tumors in models of PC, though the effects exerted by these therapies could also be related to reduced TGF-β-mediated immunosuppression and angiogenesis [62].

#### 1.1.5. Pathways of Hypoxia-Inducible Factors (HIF)

Cells react to hypoxia via the regulation of hypoxia-inducible factors (HIFs). HIF-1 is a component of the basic helix-loop-helix family of transcription factors and represents a heterodimer, composed of an alpha and a beta subunit (HIF-1a and HIF-b) [63,64]. HIF-1a is hydroxylated by HIF prolyl-hydroxylase, which then targets HIF-1a for degradation under normoxic conditions [65]. Hydroxylated HIF-1a is specifically ubiquitinated by the von Hippel-Lindau (VHL) E3 ubiquitin ligase, marking HIF-1a for proteasomal degeneration [66]. Under hypoxic circumstances, the hydroxylation of HIF-1a is restricted by the disposal of oxygen molecules and HIF-1a is secured and assembles [65]. HIF-1a can then dichotomize with HIF-b and prompt the transcription of hypoxia-survival genes [67]. Among the transcripts managed by HIF-1 is VEGF, which permits tissues to adjust to a hypoxic environment by enhancing angiogenesis.

#### 1.1.6. Cyclooxygenases (COXs)

COXs are enzymes that synthetize prostaglandins and thromboxanes from arachidonic acids and are mostly connected to inflammatory reactions [68]. Fatty acids and inflammation and their role in genitourinary cancer is an area of intensive research [69]. Even if clinical data does not clearly support a therapeutic role of non-steroidal anti-inflammatory drugs (NSAIDs) in PC, the results from multiple preclinical studies are supporting a potential therapeutic activity [70]. COXs and their eicosanoids products, prostaglandins and thromboxanes, actively participate in the regulation of endothelial cells biology [71]. There are two different forms of COXs; COX1, which is expressed constitutively and COX2, which is expressed under the influence of various growth factors and cytokines [72]. The enhanced activation of COX2 has been demonstrated in a variety of cell types in the tumor environment, and it encourages angiogenesis via increased VEGFA production, endothelial cells’ mobilization, vascular sprouting, and expanded endothelial cells’ survival [68]. COX2 has been shown to be overexpressed in PC tissue in comparison to the normal prostate tissue, and it is associated with increased MVDs in PC. Interestingly, epidemiologic research has shown a lower risk of PC in men using aspirin and other NSAIDs, which has been attributed to COX2 inhibition and therefore restrained angiogenesis [72].

#### 1.1.7. Interleukins (ILs)

ILs, which are cytokines initially produced by leukocytes, participate in the formation of the tumor microenvironment mainly via their immune controlling characteristics [73]. Together with lymphocytes, monocytes, and macrophages, endothelial cells have been reported to respond to ILs in the tissue microenvironment. To date, 50 different ILs have been identified, each binding to a unique type of receptor, having a specific origin, structure, and properties [74]. ILs maintain a variety of actions in PC, such as being molecular predictors of progression from androgen-dependent to androgen-independent PC, or behaving as tumor inhibitors [75]. They manage endothelial cells’ effects and angiogenesis in PC either in a positive or negative way. Some ILs, such as IL8, have been linked to increased PC angiogenesis, while others, such as IL27 and IL10 have been linked to angiogenesis suppression in PC [76]. IL8 expression in PC has been proven to correspond with intra-tumoral MVD [77]. Moreover, PC cells co-expressing IL8 develop rapidly in mice with enhanced tumor vascularity. Therefore, targeting IL8 in PC may lead to the development of new therapeutic for PC [74]. However, there are some ILs, such as IL27 and IL10, which have a negative effect in angiogenesis during PC development [73]. Rather than directly affecting endothelial cells, the antiangiogenic properties of IL27 are mediated through the downregulation of proangiogenic-related genes, such as *FLT1*, *prostaglandin G/H synthase 1/cyclooxygenase-1* (*PTGS1/COX-1*) and *FGFR3*s and the upregulation of antiangiogenic genes, such as *C-X-C motif chemokine ligand 10* (*CXCL10*) and *TIMP3* [78]. IL10 also has negative effect in proangiogenic cells of PC, such as activated macrophages, by obstructing proangiogenic MMP2 and overexpressing TIMP 1. Therefore, IL10 inhibits the pathway of angiogenesis in PC progression [74].

#### 1.1.8. microRNAs (miRNAs)

microRNAs (miRNA) represent stable, single stranded molecules with hairpin-loop shape and small size found in exosomes with an important role in the pathogenesis of several malignancies [79,80,81,82,83]. There is evidence that miRNAs contribute to the angiogenesis in PC [84,85]. miRNAs can regulate endothelial cells via non-cell-autonomous, as well as cell-autonomous techniques, and therefore control angiogenesis [68,86]. Both the overexpression and repression of miRNAs play a significant role on PC development and angiogenesis. While miRNAs, such as miR-296, miR-30d, miR-323, miR-21 and miR-182, are upregulated in PC, the decreased expressions of miR-195, miR-218 and miR-146a are also shown to be associated with increased angiogenesis in PC [87]. The overexpression of miR-30d and miR-323 were described to increase *VEGF* synthesis and secretion by PC cells and, thus, enhance *VEGF*-mediated angiogenesis in PC [88]. miR-296 controls the levels of VEGF and platelet-derived growth factor (PDGF) receptors in angiogenic endothelial cells, while miR-21 and miR-182 regulates the activation of HIF-1α and HIF-1α-moderated angiogenesis [89]. The reduced activation of miR-146a was described in CRPC, where it controls the expression of epidermal growth factor receptor (EGFR) and MMP2 in PC tissue [90]. On the contrary, the activation of miR-195 in PC ensues the upregulation of *ribosomal protein S6 kinase B1* (*RPS6KB1*), which results in enhanced activation of MMP-9 and VEGF proteins that play a significant role in angiogenesis. miR-218, which inhibits angiogenesis via aiming at the rapamycin-insensitive companion of mTOR (RICTOR)/VEGFA axis is also downregulated during PC development [91]. Moreover, the decreased expression of miR-130b in PC tissue is connected to a worse outcome, as the miR-130b/TNF-α/NF-κB/VEGFA loop restrains PC angiogenesis [86].

Table 1 depicts the factors that are involved in the angiogenesis in PC.

## 2. Chemotherapeutic Agents with Antiangiogenic Activity

Almost all cytotoxic agents can determine endothelial cell death if administered in very high amounts or to a low dose for a prolonged time (metronomic chemotherapy) [92]. There are some established criteria describing the characteristics of an effective antiangiogenic cytotoxic agent [93]. First, the agents should be toxic to endothelial cells at lower doses than those essential for a toxic effect on cancer cells. Second, the drugs could have an antiangiogenic effect without actually killing endothelial cells. Third, the agents must target a specific angiogenic pathway. Lastly, the antiangiogenic activity should be reproducible in vivo [94]. The taxanes are perfect examples of a family of chemotherapeutic agents which fit the above-mentioned criteria and have been researched and examined in hormone-refractory PC [95]. Docetaxel acts by inhibiting microtubule function. It is possible that PC cells can concentrate this drug intracellularly, permitting a low-dose extracellular concentration. In addition, docetaxel can bind to Bcl-2, an anti-apoptotic protein overexpressed in PC cells. Bcl-XL, another anti-apoptotic molecule, is also downregulated by docetaxel [96]. Alkylating agents such as cyclophosphamide have been used with some limited success. Regimens incorporating cyclophosphamide with doxorubicin have demonstrated a 46% PSA response [97].

## 3. Anti-Angiogenic Agents in Prostate Cancer

In this section, we discuss the most well studied antiangiogenic strategies for the treatment of PC.

### 3.1. VEGF-Directed Agents

#### 3.1.1. Bevacizumab

Bevacizumab is a humanized monoclonal antibody that recognizes all VEGF isoforms, averting connection to the VEGFR [98]. It has been used in combination with chemotherapy for the treatment of locally advanced, recurrent or metastatic non-small cell lung cancer, metastatic colorectal cancer, recurrent/metastatic cervical and ovarian/fallopian tube/peritoneal cancer, as well as for the metastatic breast cancer [only European Medicines Agency (EMA)-approved]. Moreover, it has been approved by the FDA in combination with interferon alfa for the metastatic renal cell cancer and as monotherapy for the recurrent glioblastoma. Historically, single agent bevacizumab was initially tested in 15 patients with CRPC. Bevacizumab was given at a dosage of 10 mg/kg every 2 weeks for 6 cycles [99]. There were no patients who had a 50% PSA decline and, therefore, the study was considered negative. It is important to note that antiangiogenic drugs are mainly cytostatic rather than cytotoxic; for this reason, radiographic and PSA are possibly not best approaches to assess clinical response. Interestingly, Iacobelli presented a case report of a patient with castrate-resistant PC who was treated with single agent bevacizumab when he refused chemotherapy [100]. Bevacizumab 7.5 mg/kg every 14 days was used for more than 6 months with reduction in PSA from 14 to 4 ng/mL in 1 month, as well as radiographic response of retroperitoneal lymphadenopathy.

Bevacizumab has also been evaluated when prescribed in combination with cytotoxic agents in PC. A cooperative group study, CALGB 90006, used bevacizumab in combination with docetaxel and estramustine in PC patients who were chemotherapy-naïve [101]. Docetaxel was given at 70 mg/m^2^ every 3 weeks in combination with estramustine 280 mg three times daily on days one through five plus bevacizumab 15 mg/kg on day 2 of the chemotherapy cycle. The study resulted in a 50% PSA decrease in 77% of patients. Moreover, in a phase II study of 20 patients with docetaxel-refractory mCRPC, bevacizumab was added to standard of care docetaxel [102]. Patients were treated with docetaxel 60 mg/m^2^ and bevacizumab 10 mg/kg every 3 weeks. PSA declines of ≥50% were seen in 55% of patients. A randomized phase III trial compared docetaxel plus prednisone to the merging of docetaxel, prednisone, and bevacizumab in patients who are chemotherapy-naïve [103]. This study enrolled 1050 patients with chemotherapy-naïve, mCRPC to receive docetaxel plus prednisone (docetaxel 75 mg/m^2^ on day 1; prednisone 5 mg orally twice daily) with either bevacizumab 15 mg/kg or placebo, given on day 1 of a 21-day cycle. The study failed to achieve its primary endpoint of survival, and the bevacizumab arm showed an excessive percentage of treatment-related toxicity and mortality. The rate of grade 3 adverse events in the bevacizumab arm was 74.8% compared to 55.3% in the placebo arm (*p* < 0.001). Furthermore, there was a 4.4% toxic death rate on the bevacizumab arm in contrast to a percentage of 1.1% in the placebo arm (*p* = 0.0014). The greater number of deaths caused due to treatment were infection-related. On the contrary, the bevacizumab arm exhibited superiority in PFS and rates of ≥50% PSA decrease.

#### 3.1.2. Aflibercept

Aflibercept is a fusion protein comprising the extracellular domain of the human VEGFR merged to the Fc part of human immunoglobulin G1 [104]. Aflibercept efficiently binds to VEGF-A, VEGF-B and placenta growth factors (PlGF)-1 and -2, and, in this way, it inhibits angiogenesis. It is approved by the FDA for the treatment of patients with metastatic colorectal cancer after failure of an oxaliplatin-based regimen [44]. Aflibercept has been examined in phase I and II clinical trials combined with docetaxel; however, major toxicities arose from this combination, such as hypertension, proteinuria, epistaxis, and dysphonia. VENICE is a phase III, randomized, double-blind placebo-controlled trial, involving 1224 chemotherapy-naïve patients with mCRPC, who were randomized to docetaxel, prednisone and aflibercept or to docetaxel, prednisone and placebo [105]. The study was negative because of lack of an OS advantage in the aflibercept arm. Moreover, a remarkable increase in the percentage of side effects was reported in the aflibercept arm, such as severe gastrointestinal symptoms, hypertension, bleeding, fatigue, infections, and treatment-related fatal adverse events.

### 3.2. VEGFR Tyrosine Kinase Inhibitors

#### 3.2.1. Sorafenib

Multiple tyrosine kinase inhibitors target the VEGFR. Sorafenib is a multi-kinase inhibitor that can target tumor cell proliferation via Raf kinase inhibition and angiogenesis via inhibition of VEGFR-2, VEGFR-3 and PDGF receptor kinases [106]. Phase II studies in PC have shown interesting results. Twenty-two patients with metastatic androgen independent PC were enrolled onto a phase II National Cancer Institute (NCI)-sponsored study of sorafenib 400 mg twice daily. The majority of the patients (59%) received more than one line of chemotherapy before enrolling on this study. Patients with ≥50% PSA decrease were not detected [107]. Interestingly, 2 patients with increasing PSA levels exhibited resolution of the lesions in the bone scan, suggesting that sorafenib can stimulate PSA secretion, while exerting the antitumor activity on PC. A phase II European study using sorafenib as a single agent at a dose of 400 mg twice daily has also been performed. The 55 males who took part in the trial all had chemotherapy-naïve CRPC. Among them, 2 patients had ≥50% PSA declines at 12 weeks [108]. Sorafenib could possibly have a minor clinical activity in PC as a single agent, although it still needs to be further investigated at earlier stages of the disease, as well as in combination with chemotherapy or other anti-PC treatments.

#### 3.2.2. Sunitinib

Sunitinib is a multi-tyrosine kinase inhibitor that inhibits VEGF and PDGF receptors [109]. Michaelson et al. published a phase II study, which included 34 males, half chemotherapy-naïve and half docetaxel-resistant, who were treated with sunitinib 50 mg daily for 4 weeks, followed by 2 weeks rest [110]. Among them, only one did not have metastatic disease. Two patients experienced a ≥50% PSA decrease and the best radiographic reaction was partial response in only one patient [110].

#### 3.2.3. Cediranib

Cediranib is an oral small molecule inhibitor of VEGFR-1, VEGFR-2 and VEGFR-3 and also of PDGF receptor and c-kit [111]. Cediranib has shown more promising results compared to sunitinib and sorafenib when utilized for the treatment of PC. A phase I trial described that up to 20 mg of cediranib can be used with minimal to almost no toxicity [112]. A phase II study enrolled 59 patients, 39 of whom had been previously treated with at least 2 chemotherapy combinations. Six out of the 39 patients with measurable disease had partial responses. At 6 months, 43.9% of patients were progression free; the median PFS and OS were 3.7 and 10.1 months, respectively [112]. The most common adverse events were fatigue, anorexia, weight loss and hypertension, which were dramatically decreased when prednisolone was added to the regimen. Overall, PSA increase did not correspond to change in imaging, similarly to what observed with sorafenib.

#### 3.2.4. Vandetanib

Vandetanib is an oral multi-tyrosine kinase inhibitor that targets VEGFR-2, EGFR and RET (rearranged during transfection) pathways in cancer [113]. Vandetanib was combined with docetaxel/prednisolone and studied in a phase II trial of 86 CRPC patients; no positive results were reported [114]. Moreover, in a phase II randomized trial in mCRPC patients, the addition of vandetanib to bicalutamide did not increase the activity of single-agent bicalutamide and was associated with reduced tolerability of the combination [115].

#### 3.2.5. Cabozantinib

Cabozantinib is an orally available kinase-inhibitor that targets c-MET, VEGFR, RET and other tyrosine kinases [116]. It has shown preclinical activity in a multitude of tumor types, including breast, lung, medullary thyroid, as well as PC and is also considered as a new standard-of-care first and second line treatment option for renal cell carcinoma [117,118]. Cabozantinib may also provide a new therapeutic choice for patients with radioiodine-refractory differentiated thyroid cancer, who have no available standard of care [119]. Preclinical studies have reported that it has activity in PC by preventing angiogenesis, whereas clinical studies have shown promising results in terms of bone lesion resolution, and reduction in patients’ circulating tumor cell (CTC) counts [120,121]. A phase II randomized trial of cabozantinib was prematurely terminated because of evidence of efficacy in the cabozantinib arm [122]. On the contrary, a phase III study of cabozantinib, recruiting patients with previously treated mCRPC, reported no overall improvement in the cabozantinib arm [123]. Finally, the combination of the cabozantinib with the immune checkpoint inhibitor atezolizumab achieved encouraging activity in patients with mCRPC, according to the results of COSMIC-021 [124]. This is a phase Ib, open-label study with a dose-escalation phase and a multicohort expansion phase. The dose-escalation phase determined that the optimal dose of cabozantinib is 40 mg once daily when used in combination with a 1200 mg infusion of atezolizumab once every 3 weeks. The objective response rate was 32% at a median follow-up of 12.6 months.

### 3.3. PDGF-Targeted Therapy

Imatinib is a multi-tyrosine kinase inhibitor with anti-PDGF receptor activity; it is approved for the treatment of chronic myelogenous leukemia and gastrointestinal stromal tumors. Lin et al. studied imatinib at a dose of 400 mg orally twice daily in 20 patients with non-metastatic PC and rising PSA [125]. Only a single participant had PSA decrease of ≥50% and 11 patients withdrew from the study due to toxic effects. Rao et al. also conducted a phase II study using imatinib 400 mg orally twice daily in 21 patients with biochemical only recurrence; this was an additional trial early terminated, due to unexpectedly rapid PSA rise and moderate toxicity [126]. Bajaj et al. also presented the outcome of a study utilizing imatinib 400 mg orally twice daily [127]. PSA declines of ≥50% were seen in only 2 out of 27 patients (3.7%). The majority demonstrating PSA relapse (74.1%), whilst grade 3 toxicities were reported in approximately 20% of the participants. Taking the results of the three above mentioned studies into consideration, imatinib 400 mg twice daily seems to have minimal benefit in PC.

The results of other PDGF-targeting have also been examined in patients with metastatic PC. The PDGF receptor inhibitor leflunomide was used in patients with androgen-independent PC in a phase II study of 44 men with metastatic disease. Among them, 22 had already been treated with chemotherapy [128]. Leflunomide was given intravenously with a 4-day loading dose. Three patients had PSA decrease of ≥50% and 1 of them experienced a substantial drop from 293 to 0.3 ng/mL. Therefore, there is a chance a minor subgroup of PC to have benefit from PDGF signaling inhibitors.

Lastly, imatinib has been combined with docetaxel in a randomized phase II study, enrolling 144 patients with metastatic androgen-independent PC, who received either imatinib 600 mg daily or placebo combined with 30 mg of intravenous docetaxel on days 1, 8, 15 and 22 of a 42-day cycle [129]. The percentage of a PSA decrease of ≥50%, PFS, and OS mostly leaned towards the placebo arm [130]. The study was terminated early due to increased toxicity effects, mostly of gastrointestinal origin.

### 3.4. Antiangiogenic/Immunomodulatory Drugs

#### 3.4.1. Thalidomide

Thalidomide has been approved as treatment for various hematological malignancies, including multiple myeloma. However, several studies have reported positive effects in solid tumors as well, including PC [131]. Despite its antiangiogenic characteristics not being understood in depth, numerous assays have advocated that the antiangiogenic traits could be secondary to the inhibition of secretion of two angiogenic cytokines, namely, VEGF and FGF from both tumor and stromal cells [132]. Thalidomide as a possible treatment for PC was investigated as monotherapy or combined with chemotherapy in mCRPC. A recent phase II study enrolling 63 patients compared low-dose (200 mg/day) and high-dose (up to 1200 mg/day) thalidomide; 27% of the patients experienced a decrease in PSA of ≥40% [133]. The evidence of the possible activity of thalidomide as single agent and preclinical demonstration that chemotherapy could magnify the efficacy of antiangiogenic agents constituted the basis for testing the combination of thalidomide and docetaxel in vivo [134]. A randomized phase II study enrolled 60 patients with mCRPC to receive intravenous docetaxel and bevacizumab plus oral thalidomide and prednisone. The primary end point was a PSA decrease of ≥50%. Secondary end points included PFS, OS and safety [135]. The results showed that 90% of patients receiving the combination therapy had PSA decrease of ≥50%, and 88% achieved a PSA decline of ≥30% within the first 3 months of treatment, while the side effects were manageable.

#### 3.4.2. Lenalidomide

Lenalidomide is a thalidomide derivative that mediates VEGF-mediated PI3K-Akt signaling pathway [136]. The outcomes of a phase II clinical study that treated 63 mCRPC patients with a combination of lenalidomide, docetaxel, bevacizumab and prednisone, reported that the therapeutic strategy of combining various angiogenesis inhibitors was safe and could possibly be effective [137]. Finally, in a randomized, double-blind, placebo-controlled, phase III study, 1059 chemotherapy-naive mCRPC patients were treated with lenalidomide combined with docetaxel and prednisone. However, they experienced severe adverse reactions, including hematological side effects, diarrhea, pulmonary embolism and asthenia with no apparent therapeutic benefit [138].

#### 3.4.3. Miscellaneous Angiogenesis Inhibitors

- Tasquinimod

Tasquinimod is an angiogenesis-inhibiting factor that has recently been studied as a potential treatment for PC, based on preclinical results showing activity in human PC xenograft models [139]. In a phase I study enrolling patients with mCRPC, tasquinimod showed dose-limiting side effects such as sinus tachycardia and asymptomatic amylase increases [140]. A phase II, randomized, double-blind, placebo-controlled trial of 201 patients with chemotherapy-naive mCRPC, demonstrated that oral tasquinimod resulted in a median PFS of 7.6 versus 3.3 months in those receiving placebo (*p* = 0.004) [141]. The most frequent drug-related toxic reactions involved gastrointestinal symptoms, fatigue, musculoskeletal pain and asymptomatic increase of pancreatic enzymes and inflammatory markers.

ii. Itraconazole

Another promising drug with anti-angiogenic activity is the antifungal itraconazole [142]. It has been examined in a phase II randomized trial, including 46 patients with chemotherapy-naïve mCRPC, receiving either low-dose (200 mg/day) or high-dose (600 mg/day) itraconazole [143]. There were no PSA response in the low-dose arm, a PSA decrease was seen in 14% of men in the high-dose arm, and 62% of participants changed to a favorable CTC count (<5 CTCs/7.5 mL blood) after itraconazole treatment. Interestingly, the activity of itraconazole was not consequence of androgen repression, similarly to ketoconazole [144]. The major side effects of itraconazole are fatigue, nausea, anorexia and rash, along with a syndrome consisting of hypokalemia, hypertension and edema.

iii. Trebanabib

Trebananib is an innovative peptide-Fc fusion protein that inhibits tumor endothelial cell expansion by inhibiting binding of angiopoietins 1 and 2 and the Tie2 receptor [145]. A phase I study included 37 patients and showed evidence of activity in advanced solid tumors [146]. Stable disease was observed in 7 patients, 2 of whom experienced stable disease >4 months. The reported adverse events were venous thrombosis, pleural effusion, thrombocytopenia, transient ischemic attack, and cerebral edema with headache and hydrocephalus.

**Figure 2:**
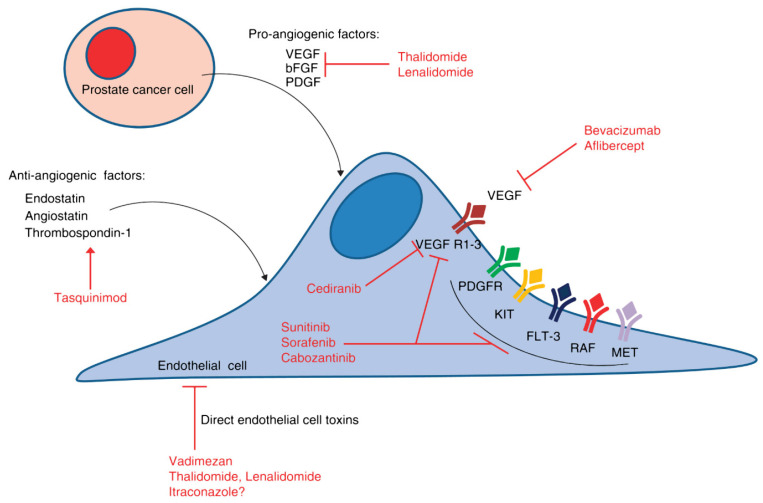
Angiogenic signaling pathways and key antiangiogenic targets in PC. The hypoxic tumor microenvironment stimulates the production of angiogenic cytokines, such as VEGF and basic fibroblast growth factor (bFGF), among others. In addition, there are naturally occurring antiangiogenic factors, such as endostatin and angiostatin, which interact with the proangiogenic factors to determine the overall angiogenic potential of the tumor microenvironment.

Table 2 summarizes the clinical trials of anti-angiogenic therapies, whereas Figure 2 provides the pathways of the angiogenesis and the key antiangiogenic targets in PC.

## 4. Mechanisms of Resistance

At first, it was thought angiogenesis inhibition comes with low risk for resistance development [147]. However, recent investigation proves the exact opposite. Trials on mouse endothelial cells isolated from human tumor xenografts, when compared to normal endothelial cells, demonstrated cytogenetic abnormalities in tumor derived endothelial cells [148]. In addition, redevelopment of tumors, while on treatment with antibodies to VEGFR-1 and -2, after an initial period of growth restriction was reported in a pancreatic islet cell tumor murine model, indicating demonstration of phenotypic resistance to VEGFR-2 blockade [149]. This resistance to VEGF blockade entails multiple plausible mechanisms, such as an eluding or inherent unresponsiveness of tumors [149]. Acquired resistance maybe caused by upregulation of alternative signaling pathways to evade the inhibited angiogenic pathway, recruitment to the tumor of bone-marrow derived proangiogenic cells or enhanced pericyte protection. On contrary, intrinsic resistance may involve among the others, angiogenesis tumor independence. A better understanding of specific mechanisms driving the progression of tumor disease on a patient basis may identify patients likely to benefit from specific antiangiogenic drugs or antiangiogenic combinations [150].

## 5. Conclusions and Future Directions

Even if targeting angiogenesis seems to be a promising treatment strategy for mCRPC, clinical results have been disappointing so far. Multiple clinical trials utilizing anti-angiogenic treatment pathways have resulted in discouraging outcomes; nevertheless, anti-angiogenic treatment is still promising and calls for future evaluation in mCRPC. Markers used to assess treatment response (PFS, PSA response) might not be suitable for assessing activity of angiogenesis inhibitors calling for more research in this setting.

The efficiency of combination strategies also remains an area of research. For instance, considering that VEGF-A causes immunosuppression by blocking dendritic cells maturation and consequently decreasing antigen presentation to T cells, antiangiogenic drugs may be used in combination with vaccine or immunotherapy agents in future clinical studies. Finally, there is rationale for combination of novel poly (ADP-Ribose) Polymerase (PARP) inhibitors with anti-angiogenic drugs. *PARP1* is involved in HIF-1α stabilization and consequently, inhibition of PARP may prevent HIF-1α accumulation that leads to hypoxic-induced apoptosis.

## Figures and Tables

**Table 1:** Factors involved in angiogenesis in prostate cancer.

Pathway	Factor	Clinical Impact	References
Vascular Endothelial Growth factors (VEGFs)	VEGFR 1, -2 and -3	Stimulators	[36,37,38,39,40,41,42,43,44,45]
Fibroblast growth factors (FGFs)	FGF-1 and -2, bFGF		[46,47,48,49,50,51]
Matrix metalloproteinases (MMPs)	MMP-2, -7 and -9		[52,53,54,55]
Transforming growth factor β (TGFβ)	TGFβ1		[56,57,58,59,60,61,62]
Hypoxia-inducible factors (HIF)	HIF-1a		[63,64,65,66,67]
Cyclooxygenases (COXs)	COX2		[68,69,70,71,72]
Interleukins (ILs)	IL8	Stimulator	[74,76,77]
	IL10 and IL27	Inhibitors	[73,76,78]
microRNAs (miRNAs)	miR-296, miR-30d, miR-323, miR-21, miR-182 and miR-130b	Stimulators	[88]
	miR-195, miR-218 and miR-146a	Inhibitors	[87]

**Table 2:** Clinical studies reporting on the activity and safety of anti-angiogenics among patients with prostate cancer.

Agent	Mechanism of Action	Phase	Primary Endpoint	Identifier
Bevacizumab	Recombinant humanized monoclonal antibody that blocks VEGF-A	II	ORR	NCT01083368
		II	PSA rFS	NCT00776594
Aflibercept	Binds to circulating VEGF-A	III	OS	NCT00519285
Sunitinib	Receptor tyrosine kinase inhibitor	II	≥30% PSA decline	NCT00879619
		II	PFS	NCT00734851
Sorafenib		II	Overall response rate	NCT00414388
		II	≥50% PSA decline	NCT00589420
Cediranib	Inhibitor of VEGFR-1, -2, and -3	II	PFS	NCT00527124
		II	PFS	NCT01260688
Cabozantinib	Inhibits VEGFRs, MET, and RET	II	PFS	NCT01428219
Thalidomide	Inhibition of VEGF, PI3K/Akt/NF-kappaB, and mTOR pathways	II	ORR	NCT00307294
Lenalidomide	Inhibition of VEGF-induced PI3K/Akt pathway signalling	II	OS	NCT00942578
Tasquinimod	*S100A9* protein that inhibits *VEGF*	III	PFS	NCT01234311
Itraconazole	Inhibition of the Hedgehog pathway	II	≥30% PSA decline	NCT01450683

VEGF-A: vascular endothelial growth factor; ORR: objective response rate; PSA rFS: PSA recurrence-free survival; OS: overall survival; PFS: progression free survival; VEGFR: vascular endothelial growth factor receptors; PI3K/Akt NF-kappaB: phosphoinositide 3 kinase/protein kinase B/nuclear factor-kappa B; mTOR: mammalian target of rapamycin.
